# Transcriptome analysis of flower bud identified genes associated with pistil abortions between long branches and spur twigs in apricots (*Prunus armeniaca* L.)

**DOI:** 10.1371/journal.pone.0273109

**Published:** 2022-08-26

**Authors:** Qiu-ping Zhang, Xiao Wei

**Affiliations:** Liaoning Institute of Pomolgy, Yingkou, Liaoning, China; Institute for Horticultural Plants, China Agricultural University, CHINA

## Abstract

Pistil abortions of flower buds occur frequently in many apricot cultivars, especially in long branches. However, the molecular mechanism underlying pistil abortion in apricots remains unclear. To better understand the molecular mechanism of pistil abortions between long branches and spur twigs, paraffin sections and high-throughput sequencing technology were employed to analyze the expression patterns of genes associated with pistil abortions during later flower bud development stage in ‘Shajinhong’ apricot. The result of stage III (separation of bud scales) was the critical stage of pistil abortion in apricots. A total of 163 differentially expressed genes were identified as candidate genes related to pistil abortion in long branches. These genes are implicated in programmed cell death, hormone signaling, cell wall degeneration, and the carbohydrate metabolism pathway. The results showed that the up-regulation of gene expression of Xyloglucan endotransglucosylase/hydrolase and β-glucosidase in flower buds might be the direct cause of cell wall breakdown and pistil necrosis in long branches. We hypothesize that there is a molecular relationship between pistil abortion before blooming and cellulose degradation, and then carbohydrate transport in the case of carbon deficiency in long branches. Our work provides new insights into cellulose degradation in abortion pistils and valuable information on flower development in apricots, and also provides a useful reference for cultivation regulation in apricot or other fruit crops.

## Introduction

Floral organ abortion is the result of nutrient competition between plant survival and reproduction, and it has been found in many domesticated plants [1–3]. Apricot is a crop for harvesting fruit; therefore, pistil abortion determines fruit set and stable yield potential [4]. After dormancy, both vegetative growth and reproductive growth of perennial plants require a lot of carbohydrate nutrients from other tissues [5, 6], which forms a special competition pattern between them. Therefore, it is essential to understand the molecular mechanisms of the competition patterns.

Although apricot is a fruit tree that forms flower buds very easily, many cultivars produce a large number of pistil-abortive flowers during blossoming, especially on their long branches [4, 7]. Pistil-aborted flowers have no ability to fertilize and fall off after flowering, affecting the yield of apricot fruit [8]. Pistil abortion in apricots is a common phenomenon, including pistils below the stamens, withered pistils, or an absence pistil [6, 8, 9]. In fact, many cultivars have reported pistil abortion [6, 7, 9, 10]. Two time waves of the pistil abortion were identified during the stages from mid-November to mid-December and from mid-February to mid-March of the following year in the kernel-using apricot [11]. Pistil abortion appeared at the late stage of pistil differentiation in the ‘Lanzhou Dajie’ apricot [8]. No obvious pistil abortion of flower buds was observed during the physiological differentiation stage in ‘Yubada’ apricot; however, a large number of pistil abortion flowers still appeared after flowering, and Kong [10] speculated that pistil abortion might occur in the calyx stage of flower buds. In brief, flower development in the late stage usually determines a well-developed pistil during blossoming. However, the critical timing of pistil abortion after dormancy remains unclear.

Generally, the flower bud differentiation of primordia of each organ is normal in the early stage. Still in the subsequent development process, organ development is affected by various factors resulting in pollen dysplasia [12] and pistil stagnation or necrosis [7, 10]. The study of pistil abortion in apricots mainly focused on heredity [9, 13] and temperature [5, 6, 8]. According to an investigation of anomalous flowers, the pistil abortion rate of Chinese apricot cultivars was significantly higher than that of European apricot cultivars, and the pistil abortion rate of some cultivars was 96.2% [9]. Under the same environmental conditions, the proportion of normal flowers of ‘Longwangmao’ and ‘Baiyubian’ cultivars were 91.8% and 41.2%, respectively, indicating that differences in genotypes were the main reason for the difference in pistil development [14]. Temperature is an important factor in floral organ development. Viti [15] found that a higher temperature in dormancy leads to abnormal metabolism of flower bud cells and increases the pistil abortion rate. Low temperature after dormancy has an effect on the normal development of ovular nucellus in apricots [8], and low temperature before blooming causes stagnant pistil or atrophy [11]; however, higher temperatures before flowering might also increase the pistil abortion rate [5]. Despite the same genotype and environmental conditions, the pistil abortion rate of flower buds in long branches was significantly higher than that in spur twigs in apricots.

There have been a few studies on the effects of pistil abortion on endogenous nutrient storage or hypotrophy in apricots [5, 16]. Julian [4] found that the weight of flower buds from short branches was significantly heavier than from long braches during bud initiation and the following stages of development before blooms. There was a significant difference in the weight/quality of flower buds, which might be related to the distribution of carbon storage or transport in the tree. Starch flower buds were hydrolyzed rapidly during flower bud differentiation, and soluble sugar content increased rapidly [17]. High flower anomalies were directly related to the low photosynthetic output of leaves and low allocation ratio to flower buds based on 14C isotope tracking [16]. The main factors leading to the pistil abortion of long branches may be related to the catabolism of macromolecule nutrients in the flower bud [2]. Glucose metabolism, starch metabolism, and photosynthesis were also related to pistil abortion in Japanese apricots [17]. In addition, boron ions could mainly improve fruiting ability by acting on fertilization in spur twigs and had little effect on long branches in apricots [7]. In summary, studies on pistil abortion in apricots are mainly limited to the physiological and biochemical changes observed by cytology, such as tissue sections and morphological features; however, the molecular mechanism involved in pistil abortion remains unknown.

Although pistil abortion and carbohydrate balance in different types of branches have been reported, few studies have focused on the molecular mechanism and transcriptional expression of genes related to pistil abortion in apricots. ‘Shajinhong’ apricot with large fruit and good quality is one of the most famous local cultivars in China. However, pistil abortion rate is as high as 72.2%, which seriously affects the normal fruit setting and yield. To clear the mechanisms of pistil abortion in ‘Shajinhong’ apricot, in this study, our work conducted the transcriptome sequencing analysis of spur twigs and long branches based on the cytological characteristics of the flower buds. This result can help us to understand the mechanisms of pistil abortion, identify the gene regulatory networks involved in the determination of pistil development, and provide a useful reference for cultivation regulation and breeding in apricots.

## Materials and methods

### Plant material

Eight years old trees of apricot ‘Shajinhong’ with the space of 5×5 m were planted in an orchard in the Liaoning Institute of Pomology. ‘Shajinhong’ originated in Donggang County of Liaoning province, and introduced into Chinese Germplasm Repository for Plums and Apricots in 1985. This cultivar is of top quality, a productive one with an attractive appearance for fresh or processing market, and it is a rare firm flesh cultivar among many Chinese local cultivars [18]. However, its pistil abortion rate is as high as 72.2%, which seriously affects the normal fruit setting and yield. Two types of branches of the current year were selected: spur twigs or short branches (SD), between 5 and 15 cm in length, and long branches (SC) longer than 1 m and with a basal diameter greater than 1.5 cm. The flower buds per biological replicate were randomly sampled around the canopy from the same tree of each replicating at a height of 1.5~2.0 m.

**Fig 1:**
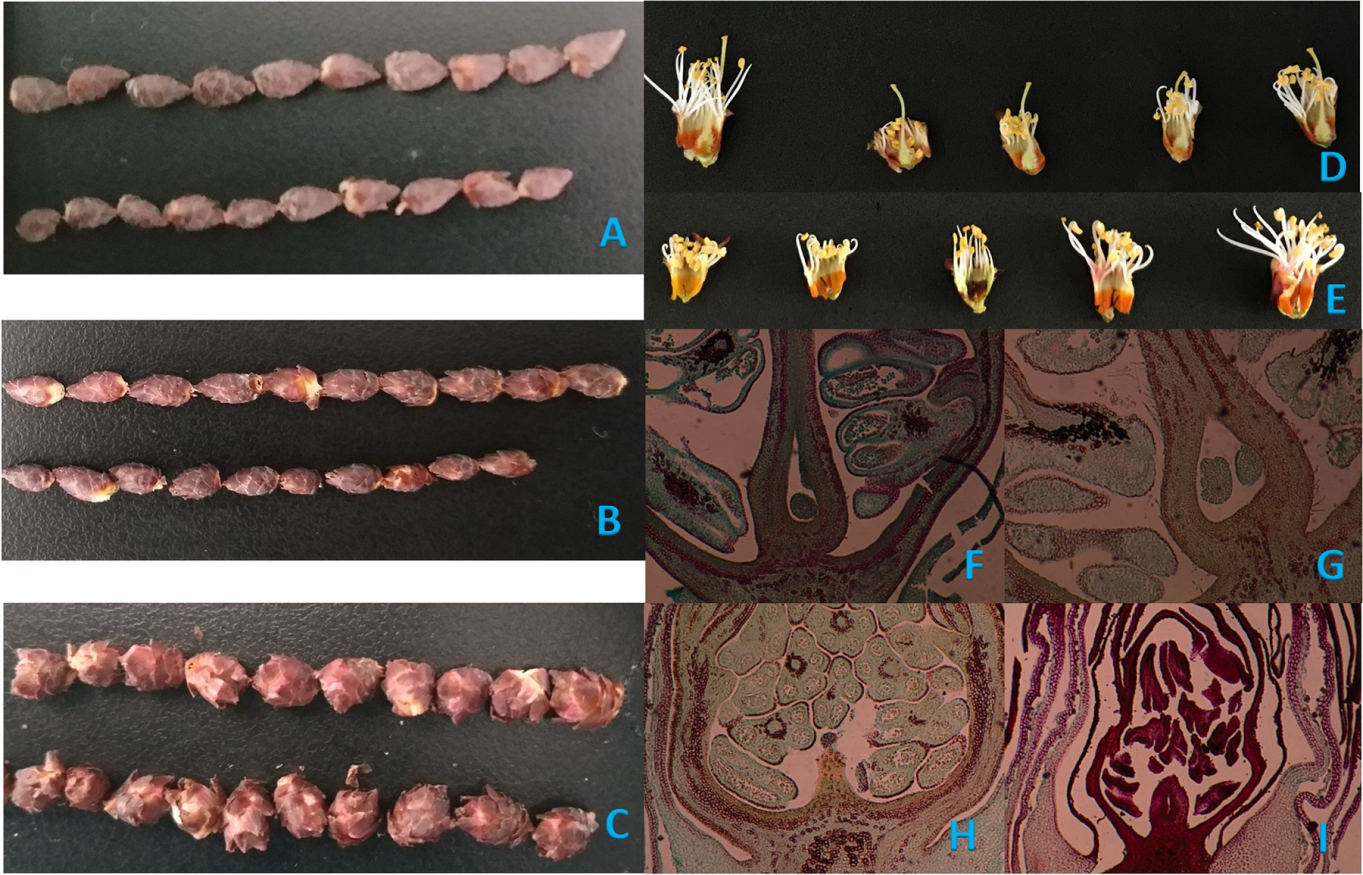
Morphology of flower buds of ‘Shajinhong’ apricots. (A) The features of flower bud of spur twigs (up) and long branches (down) at stage I. (B) The features at stage II. (C) The features at stage III. (D) The features of normal flowers. (E) The features of aborted flowers. (F) and (G), the vertical section of normal flower bud. (H) and (I), the vertical section of aborted flower bud.

According to Austin and Hewett’s criteria for flower bud development [19], we randomly collected approximately 200 flower buds in different branches from the median-apical portion at the end of the dormancy stage (stage I: no visible bud growth), budding stage (stage II: visible bud growth), and descaling stage (stage III: separation of bud scales) of flower buds in 2020 (**Fig 1**). Each individual was used as a replicate, and there were three biological replicates. First, 15 flower buds were fixed in FAA [5% formalin, 5% acetic acid, 90% ethanol (v/v)] at room temperature for further use, after which the remaining buds from the same treatment were transferred immediately to liquid nitrogen and stored at -80°C for RNA extraction.

### Paraffin sections and observing

Flower buds were prepared using paraffin sections according to the methods described in [6]. After dehydration through an ethanol alcohol series, the material was embedded in paraffin blocks and longitudinally sectioned using a Leica RM2235 Rotary slicer (Leica Germany). The slices were de-waxed, rehydrated, and stained with a double staining method of saffron and solid green. The thickness of the paraffin sections was 10 μm, and the slices were observed using a NiKon Y-FL optical microscope (NIKON Japan).

### RNA extraction and RNA sequencing

Total RNA was extracted from flower buds of different branches or twigs using TRIZOL Reagent (St. Louis, Missouri, USA) according to the manufacturer’s instructions (Invitrogen, Waltham, USA), and was treated with DNA probes following RNase H digestion to remove rRNA. RNA concentration and purity were measured using NanoDrop 2000 (Thermo Fisher Scientific, Wilmington, DE). RNA integrity was assessed using the RNA Nano 6000 Assay Kit of the Agilent Bioanalyzer 2100 system (Agilent Technologies, USA). The pure mRNA was fragmented and reverse transcribed for cDNA synthesis.

For each treatment, the samples were collected for each stage with three biological replicates. The 18 RNA-seq transcriptome library preparation and sequencing of the flower buds were conducted by the Biomarker Technologies Company (Beijing, China). A total amount of 1 μg RNA per sample was used as input material for the RNA sample preparations. Sequencing libraries were generated using NEBNext ^®^ Ultra™ RNA Library Prep Kit for Illumina ^®^ (NEB, USA), following the manufacturer’s recommendations. Then, the library preparations were sequenced on an Illumina Hiseq 2000 platform (NEB, USA), and paired-end reads with fragments of approximately 240 bp in length were generated. The raw data were deposited into the NCBI Sequence Read Archive (SRA) under the accession number PRJNA767173.

### Reads mapping and gene functional annotation

Clean reads were obtained by removing reads with adapter, ploy-N, and low-quality reads (the percentage of reads with quality value 10 was more than 20%) from raw data using SeqPrep and Sickle with default parameters. The Q20, Q30, GC content, and sequence duplication level of the clean data were calculated. All down-stream analyses were based on high-quality clean data. These clean reads were separately mapped to the reference genome sequence [20] using the Hisat2 software. Only reads with a perfect match or one mismatch were further analyzed and annotated based on the reference genome. Finally, gene function was annotated based on seven known protein databases: Nr, Nt, SwissProt, InterPro, KOG, KEGG, and GO with a cutoff E-value < 10^−5^.

To identify DEGs among different samples, the expression level of each transcript was analyzed using FPKM. Sequences were considered to be significantly differentially expressed when FDR<0.001 and |Log2FC (fold change) | ≥1.8 by the DEGseq package [21]. GO functional enrichment analysis of the differentially expressed genes (DEGs) was implemented by the GOseq R packages based on Wallenius non-central hyper-geometric distribution [22], which can adjust for gene length bias in DEGs. KEGG pathway analysis was performed on the differentially expressed genes (DEGs). We used KOBAS software [23] to test the statistical enrichment of DEGs in the KEGG pathways. The top 100 functional proteins with the highest credibility were constructed using STRING. The weighted correlation network analysis (WGCNA), differential gene Venn diagram, and gene co-expression trend analysis were all performed using BMKCloud.

### Real-time qRT-PCR analysis

Total RNA was extracted and used to synthesize cDNA using PrimeScript™RT Kit (Cat. RR047A, TaKaRa, Japan). The cDNA was diluted five times and then used as a template. Quantitative real-time PCR was performed using HiScript II Q RT SuperMix and 2 × ChamQ Universal SYBR qPCR Master Mix (Vazyme, Nanjing) on an ABI 7500 Real-Time PCR Detection System (Applied Biosystems, US) with the thermocycle: 95°C, 3 min; 95°C, 15 s, 59°C, 15 s and 72°C, 20 s for 40 cycles. The actin (ACT) gene was used as an internal reference, and relative expression was calculated using the 2^-ΔΔCt^ method. Each sample was analyzed using three independent biological replicates and three technical replicates. All of the primers used in this study are listed in the S1 Table. The analysis of variance (ANOVA) was based on Fisher’s Least Significant Difference (LSD) in Origin 9.0 (OriginLab Corporation, US).

## Results

### Morphology characteristics of flower bud between long branches and spur twigs

The phenological characteristics of long-shoot flower buds (SC) and spur-twig flower buds (SD) were consistent in 2020. The end of the dormancy stage of lower buds (stage I: no visible bud growth) was on March 17. The budding stage (stage II: visible bud growth) was on March 24, the descaling stage (stage III: separation of bud scales) was on April 1, the balloon stage (initial protrusion of petals) was on April 3, and the opening was on April 7. As shown in Fig 1A–1C, bud growth showed an increasing trend in both types of branches from stage I to stage III, and the flower buds of long branches were visually smaller than spur twigs during the entire swollen process.

Two types of flower bud development were observed at different developmental stages to determine the critical stage of pistil abortion. The morphologies of the abortive flower and the normal developmental flower are shown in Fig 1D and 1E. From the bud vertical section, the abortive rates of flower buds in long branches and spur twigs were similar at stages I and II: 6.67% and 13.33%, respectively. It was not until stage III that there was a significant difference in the abortive rate between the two. The abortion rate of flower buds on long branches was 33.33%, while that on spur twigs was only 13.33% (**Table 1**). In the balloon stage, a large number of aborted flowers (pistils below the stamens, withered pistils, or an absence of pistils) appeared on the flower buds of long branches, which was significantly different from that on the spur twigs. The abortive rates of long branches and spur twigs were 83.19% and 42.86%, respectively. Therefore, the critical stage of pistil abortion was at stage III (separation of bud scales) in ‘Shajinhong’ apricots.

**Table 1:** The pistil abortion rate at different stages of flower buds development.

	I stage	II stage	III stage	Balloon stage
SC	6.67±0.62	13.33±0.47	13.33±0.85	42.86±2.59
SD	6.66±0.85	13.33±1.03	33.33±1.65	83.19±4.08

### Transcriptomic overviews of flower bud between long branches and spur twigs

In this study, a total of 4.0 × 108 clean reads were obtained from 18 mRNA samples on the Illumina platform, and approximately 22.2 million reads for each profile. Each stage of sample had approximately 6.6 ± 0.7 Gb of each sequencing data, and 119.45 Gb of data was measured. The Q30 percentages of the clean data for all samples ranged from 93.68% to 95.26%, and the mean GC content for all samples was 46.58%. Approximately 93.85% of clean sequence reads were mapped to the apricot genome and ranged from 93.49% to 94.40%. Among them, the percentage of clean reads that were mapped at the unique location of the reference genome was higher than 89% (Table 2). The numbers of forward and reverse mapped reads were roughly similar in all samples. Overall, the above values indicate that the sequences in this study were of quality, and the data were accurate.

**Table 2:** Statistics on the quality and mapped.

Samples	Clean reads	Clean bases (bp)	GC Content (%)	Q20 (%)	Q30 (%)	Mapped reads (%)	Uniqe mapped reads (%)
SC1-1	26,888,271	8,036,329,144	46.88	98.38	94.82	94.35	89.22
SC1-2	21,355,134	6,383,872,062	46.62	98.30	94.66	94.25	90.45
SC1-3	20,520,586	6,134,536,846	46.75	98.34	94.74	94.30	91.07
SC2-1	26,113,395	7,809,597,114	46.52	97.97	94.09	93.68	89.52
SC2-2	22,286,844	6,666,430,524	46.49	98.02	94.11	93.78	89.23
SC2-3	21,190,897	6,336,277,656	46.55	98.03	94.20	93.60	89.14
SC3-1	21,783,424	6,518,286,086	46.58	97.91	93.93	93.61	90.03
SC3-2	27,091,837	8,088,786,930	46.78	97.99	94.19	93.67	89.99
SC3-3	21,856,715	6,536,669,372	46.60	97.95	93.99	93.49	89.52
SD1-1	22,267,202	6,657,125,798	46.43	98.24	94.59	94.12	91.00
SD1-2	21,428,574	6,407,753,760	46.27	98.57	95.26	94.34	91.49
SD1-3	20,531,765	6,134,779,168	46.57	98.40	94.89	94.40	91.09
SD2-1	22,616,083	6,763,814,792	46.50	98.00	94.11	93.54	90.08
SD2-2	21,628,175	6,466,241,400	46.40	98.00	94.07	93.71	90.38
SD2-3	20,599,759	6,162,042,434	46.42	98.05	94.18	93.73	90.19
SD3-1	20,690,248	6,189,445,112	46.69	97.80	93.68	93.49	89.68
SD3-2	19,285,699	5,735,091,982	46.69	98.00	94.16	93.73	89.88
SD3-3	21,486,161	6,420,662,706	46.63	97.95	94.04	93.49	89.31
Total	399,620,769	119,447,742,886	46.58	98.11	94.32	93.85	90.07

According to the reference genome sequence of apricots (Chuanzhihong), the mapped reads were spliced using StringTie software and compared with the original genome annotation information. By filtering out sequences encoding too short peptide chains (less than 50 amino acid residues) or containing only a single exon, 32 287 transcripts were identified. It is necessary to optimize the gene structure of the reference annotations because of the boundary error upstream or downstream of the gene, and 480 genes were optimized (S2 Table). In the previously unannotated transcriptional regions, 1 714 new genes were discovered, of which 1 516 were successfully aligned to nine major functional databases for annotations (S3 Table).

A total of 32 287 genes were annotated with seven major functional databases, including information on protein sequence similarities. In the NR (non-redundant protein database, NCBI), GO (Gene Ontology), KOG (Clusters of Orthologous Groups), KEGG (Kyoto Encyclopedia of Genes and Genomes), COG (Clusters of Orthologous Groups of proteins), SwissProt and eggNOG databases, 29 253, 17 170, 14 601, 10 119, 9 552, 19 804, and 25 492 genes were aligned, respectively.

**Fig 2:**
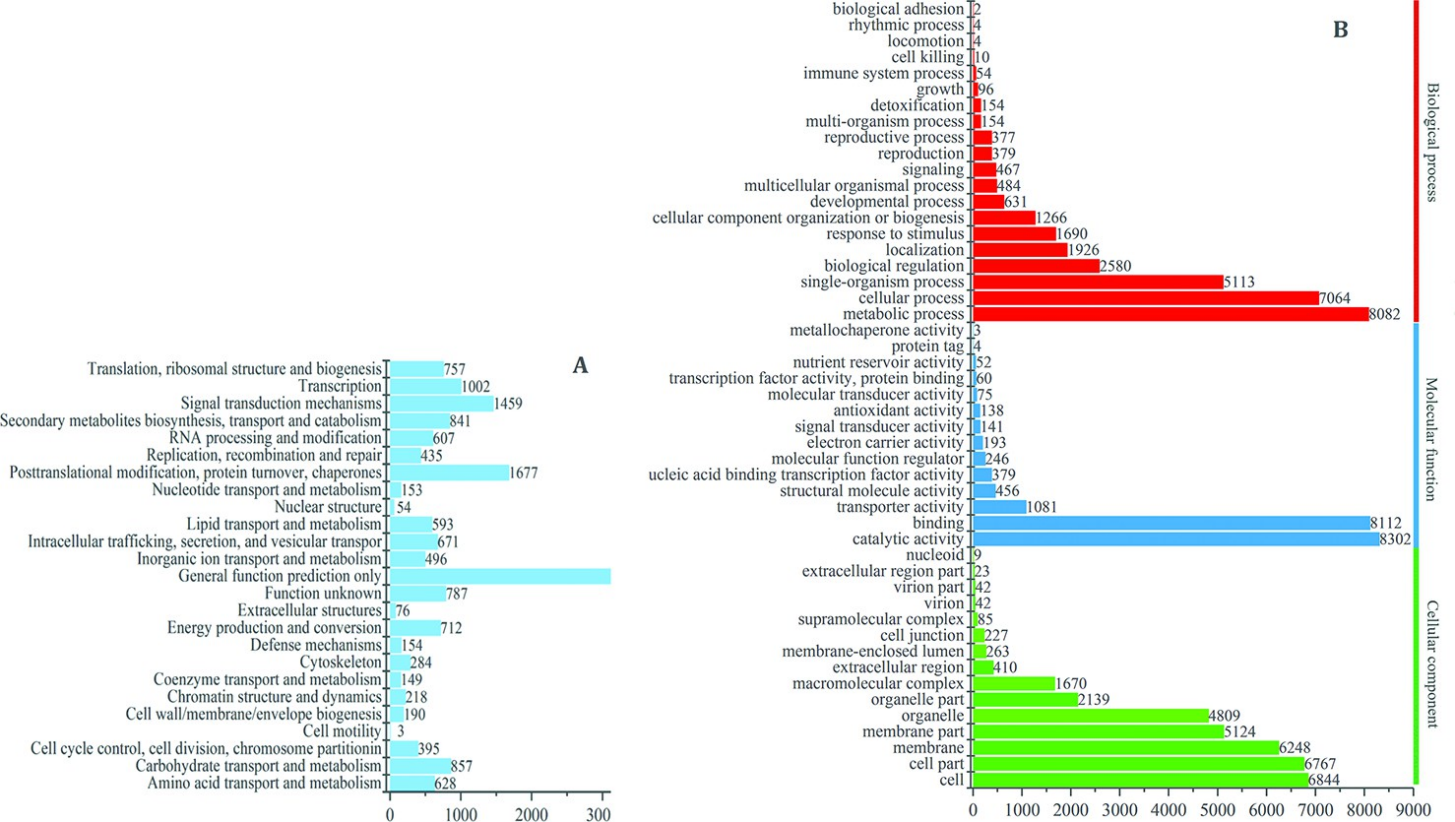
Annotation of all expressed genes of ‘Shajinhong’ apricots. (A) KOG function classification of all genes. (B) Gene ontology classification of all genes.

To further evaluate the completeness of our transcriptome libraries and the effectiveness of annotation, the annotated sequences for genes with KOG classifications were randomly searched. Among the 18 samples, 16 602 expressed genes had a specific protein function definition, accounting for 56.61% of the total annotated genes and involving 25 KOG functional classes. Among these categories, the cluster for “general function prediction only” (3 404, 20.5%) represented the largest group, followed by “posttranslational modification, protein turnover, chaperones” (1 675, 10.10%) and “signal transduction mechanisms” (1 381, 8.32%). The following three categories, “extracellular structures” (75, 0.45%), “nuclear structure” (54, 0.33%), and “cell motility” cell motility’ (3, 0.01%), accounted for the lowest percentages (Fig 2A). A total of 88 896 sequences were assigned to 47 GO terms, which could be classified into three main categories: biological processes, cellular components, and molecular functions. In each of the main GO classifications, the “metabolic process,” “cell,” and “catalytic activity” terms were significantly overrepresented. A high percentage of genes from the “cellular process,” “cell part,” and “binding” categories were also noticed, but a small percentage of genes were from “biological adhesion,” “nucleoid,” and “metallochaperone activity” (Fig 2B). Additionally, 1 528 transcription factors were predicted, and were grouped into 19 types according to the DNA binding function regions. The bHLH, bZIP, MADS, MYB, WRKY, NAC, and AP2/EREBP types contained 107, 49, 79, 175, 61, 119, and 120 genes, respectively. The expression of these TFs is related to flower development, senescence, or environmental stress.

### Expression of DEGs between long branches and spur twigs libraries

**Fig 3:**
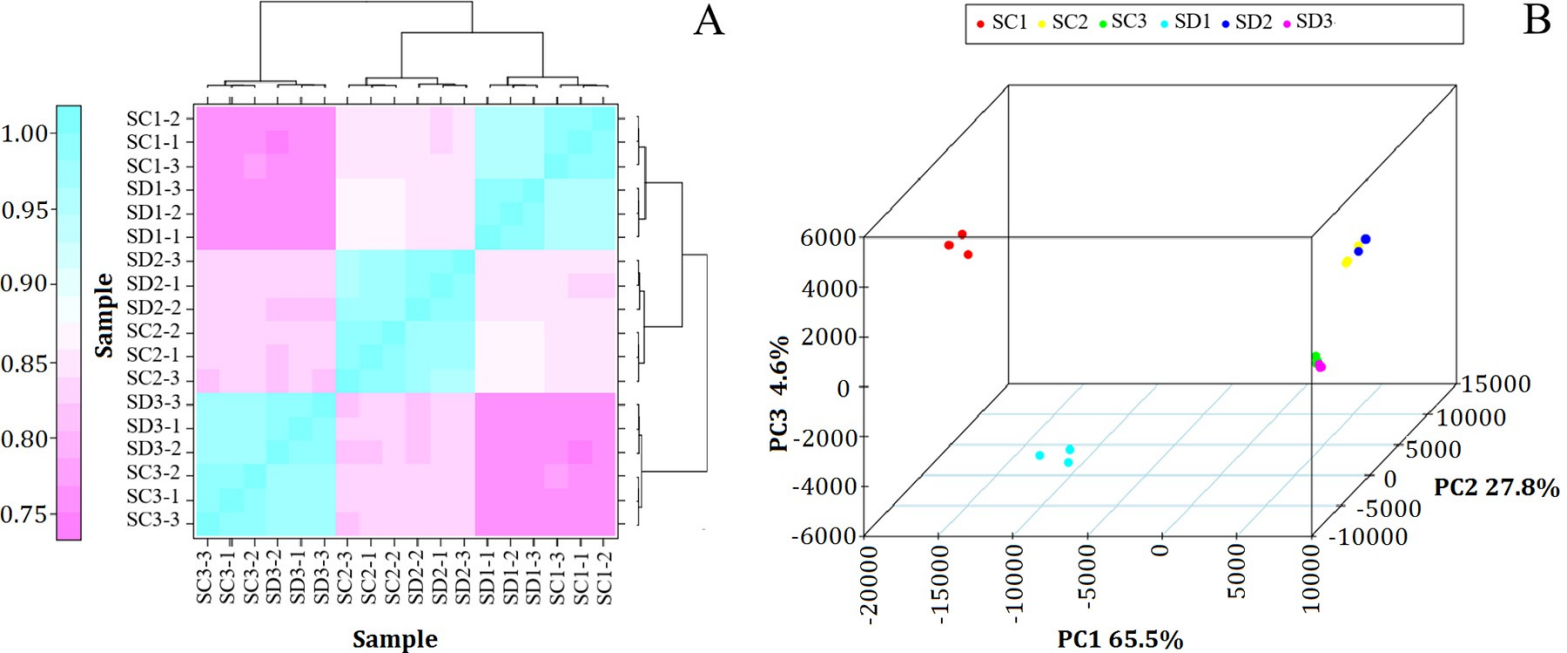
Principal component analysis (A) and cluster analysis (B) of transcriptome data. Three biological replicates per sample were analyzed for each development stage. The percentages on the axes indicate the values explained by each PCA; and the dots of the same color represent the transcriptomes of the samples obtained from the same shoot type at the same time.

Cluster analysis and principal component analysis (PCA) of the 18 mRNA samples (Fig 3) was performed using RNA-seq data. The results revealed no differences between the three replicates at each developmental stage of flower buds.

A weighted gene co-expression network analysis (WGCNA) was performed using all expression genes with fragments per kb per million (FPKM) value > 1. Genes were comprised of six co-expression modules, of which the turquoise module showed a significant association with the rate of pistil abortion during flower bud developments (S1 Fig). This module contained 1732 genes that were involved in the different stages of long branches and spur twigs (r > 0.86).

**Fig 4:**
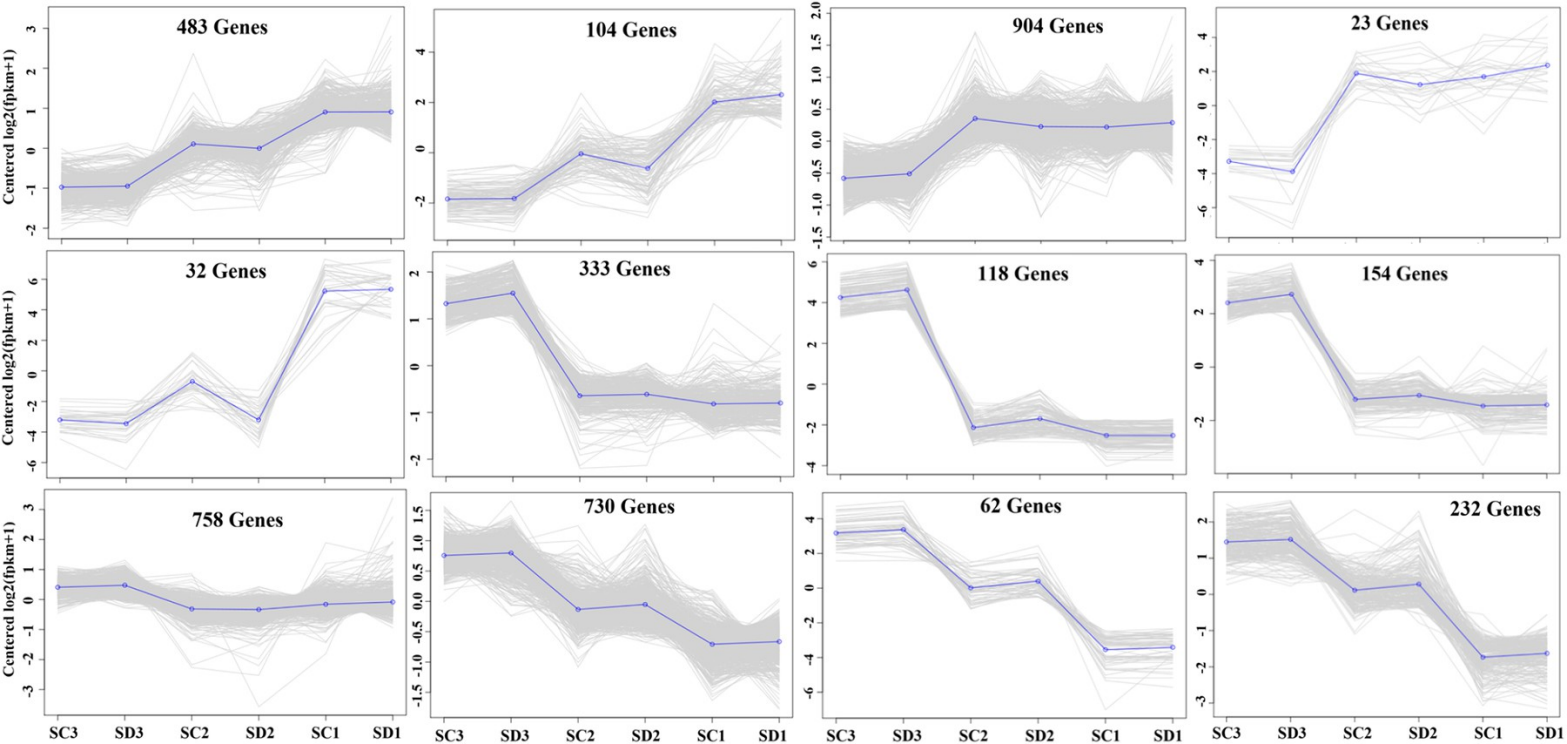
Twelve groups of gene co-expression trend of differentially expressed genes (DEGs) among three stages of flower bud development.

A total of 5 197 differentially expressed genes (DEGs) were identified at late developmental stages of flower buds with false discovery rate (FDR) ≤ 0.01 and FPKM > 1.8 in the compared libraries. The results of the gene co-expression trend analysis showed that the DEGs were divided into 17 groups, in which the gene expression of two groups (483 and 104) gradually decreased during flower bud development, and the gene expression of the three groups (including 730, 232, and 118) gradually decreased. The gene expression of 4 groups suddenly increased in stage III, the gene expression of 2 groups suddenly decreased in stage III, and the gene expression of one group decreased (Fig 4). However, the differential genes of flower buds of long branches and spur twigs in the three stages of flower bud development were 407 (at stage I), 639 (II), and 213 (III), respectively.

**Fig 5:**
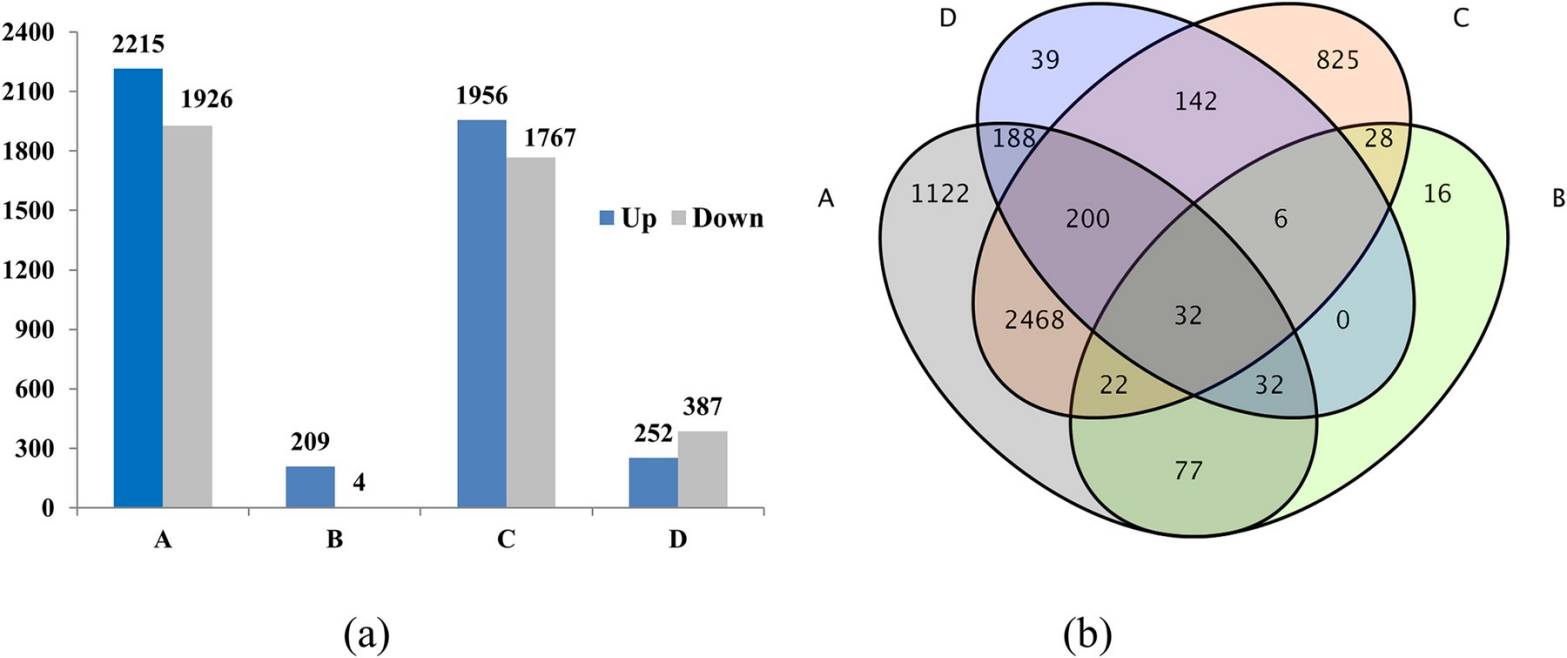
Comparison (a) and Venn diagram (b) of DEGs between long branches and spur twigs. A, SC2vsSC3; B, SD3vsSC3; C, SD2vsSD3; D, SD2vsSC2.

**Fig 6:**
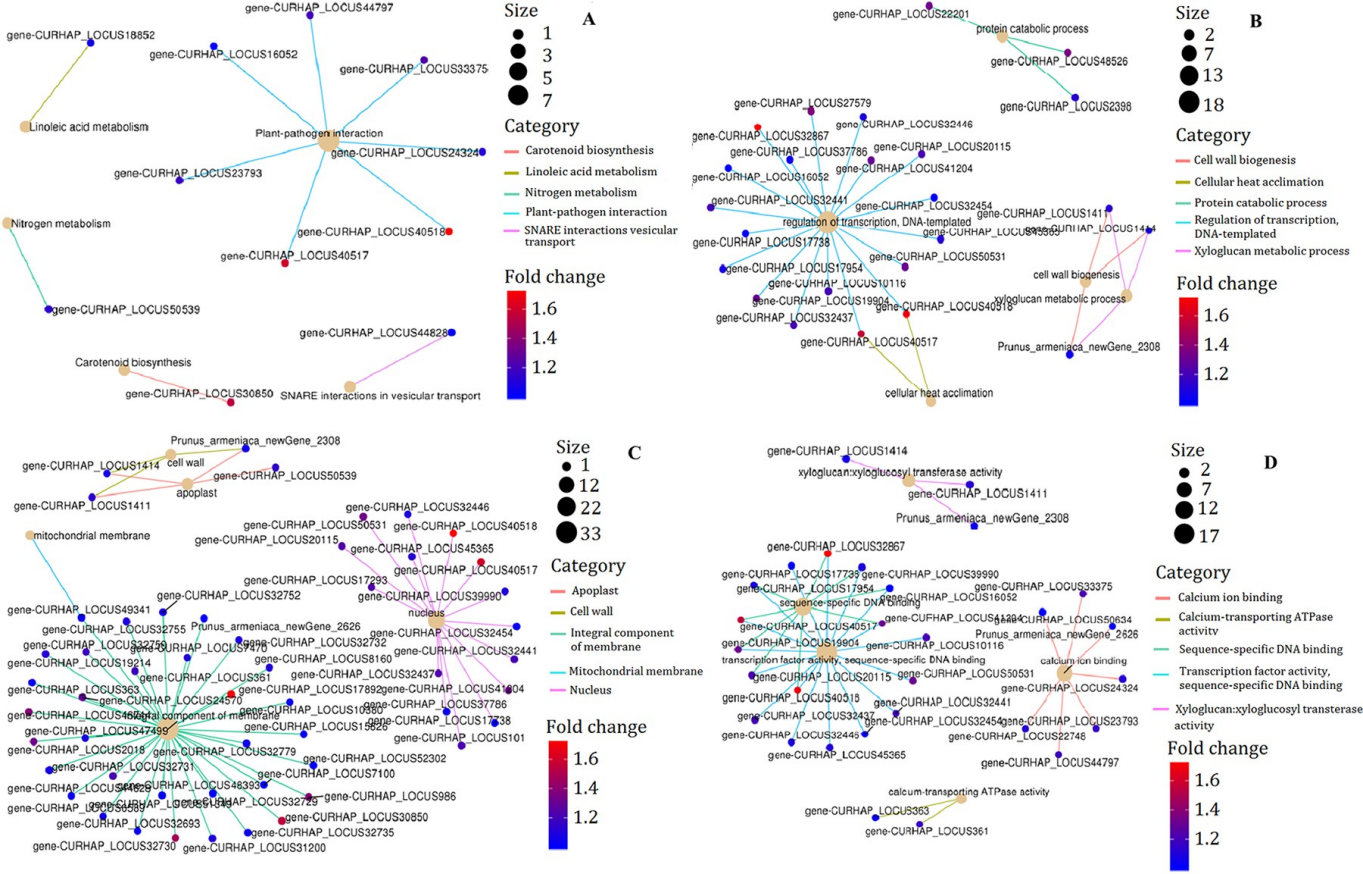
Enrich center plot of DEGs between long branches and spur twigs at Stage III. (A) KEGG. (B) Biological process component of GO classify. (C) Cellular component of GO classify. (D) Molecular function of GO classify. Red indicates high expression; blue indicates low expression; the color of lines indicates different pathways, and the color of gene nodes indicates fold of difference. The larger the nodes represents the more genes are enriched into this pathway.

A total of 4 141, 213, 3 723, and 639 showed differential expression between SC2vsSC3, SD3vsSC3, SD2vsSD3, and SD2vsSC2, respectively (Fig 5A). In stage II, there were 639 DEGs between long branches and spur twigs, of which 252 genes were significantly upregulated and 387 genes were downregulated. In stage III, there were only 213 DEGs between long shoot and spur twigs libraries, among which 209 genes were upregulated and 4 genes were downregulated. A total of 163 genes overlapped in the differential expression analysis of SD3vsSC3 (A) and SC2vsSC3 (B) in the Venn diagram (Fig 5B), and these DEGs were involved in multiple metabolic pathways. According to the functional notes of the KEGG database, many DEGs were enriched in the “plant-pathogen interaction,” “nitrogen metabolism,” and “linoleic acid metabolism” pathway (Fig 6A). The 32 genes were involved in signal transduction processes related to plant hormones or transcription factors (TFs), and 51 genes were related to physiological responses such as cell stress resistance or aging, 18 genes were related to plasma membrane composition or material transport, and 35 genes were related to biological metabolism such as carbohydrate metabolism or secondary metabolism, and the remaining 27 unknown function genes might be involved in multiple metabolic pathways (S4 Table).

Based on a comparison against the GO database, 163 DEGs between long branches and spur twigs were assigned to three main categories. In biological processes, the “regulation of transcription, DNA-templated,” “cell wall biogenesis,” and “cellular heat acclimation” were significantly overrepresented (Fig 6B). In the cell component, “integral component of membrane” represented the largest group, followed by “nucleoid,” and “cell wall” (Fig 6C). In molecular analysis, DEGs with similar functions were clustered in “transcription factor activity and sequence-specific DNA binding,” “Calcium ion binding,” “xyloglucosyl transferase activity,” and “calcium transporting ATPase activity” were closely related to cell membrane transportation (Fig 6D).

### DEGs involved in hormones or transcription factors

**Fig 7:**
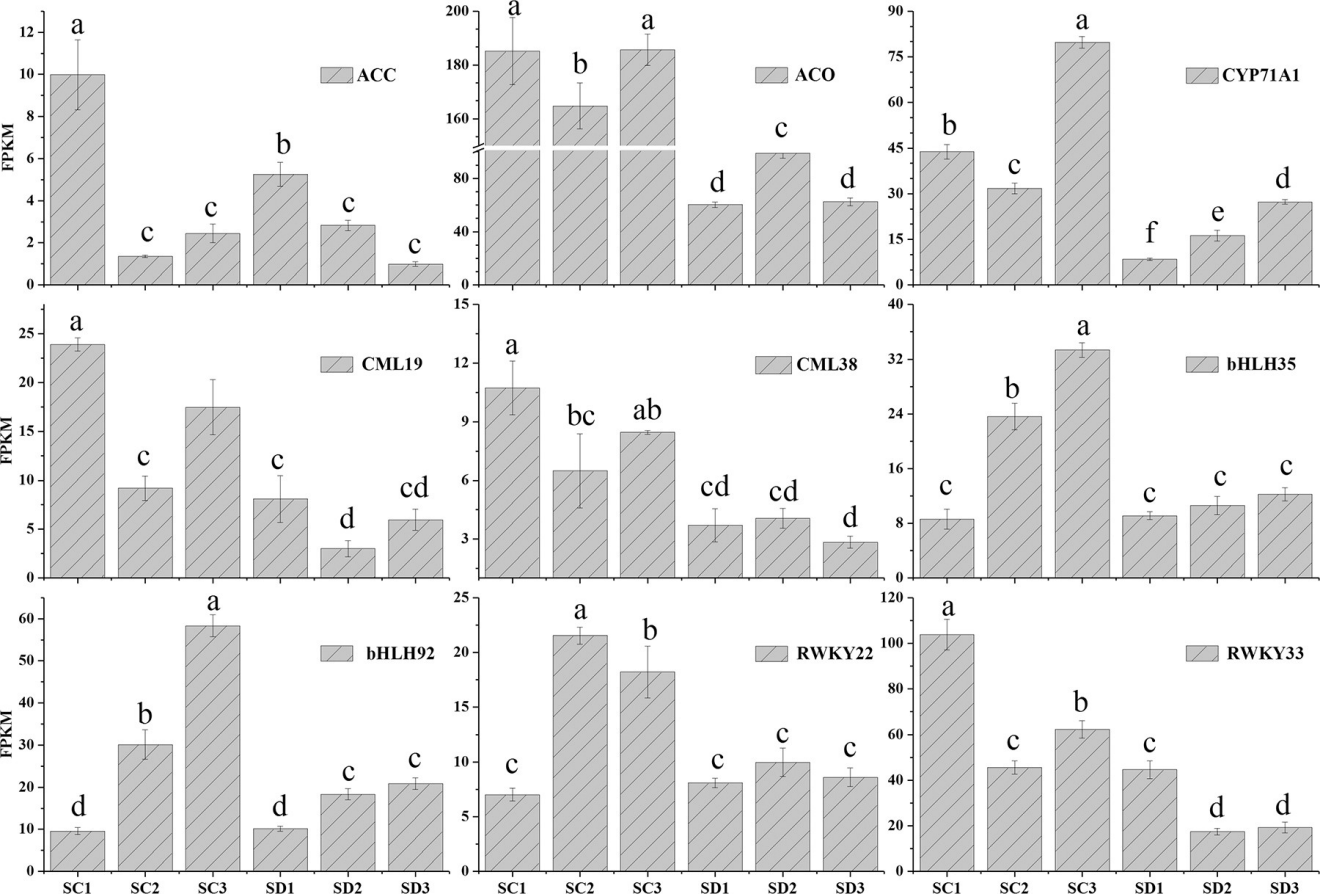
Expression analyses of genes related to hormones or transcription factors. The x-axis indicates the different stages. The y-axis shows the expression values of the FPKMs. All values were repeated three times and the data are presented as the mean±SD. Different letters indicate significant differences at P<0.01. ACC: CU_locus32671; ACO: CU_locus22526; CYP71A1: CU_locus30850; CML19: CU_locus33375; CML38:CU_locus24324; bHLH35: CU_locus1151; bHLH92: CU_locus476; WRKY22: CU_locus16052; WRKY33: CU_locus40517.

Pistil abortions, which are involved in programmed cell death (PCD) [24], are highly complex phenomena that are caused by a series of biological processes, including many genes acting synergistically, collaborating in regulating various pathways. Among them, hormones and transcription genes play two important roles in regulating organ senescence and PCD [24]. In our database, seven genes related to ethylene (ETH) metabolism, including encoding 1-aminocyclopropane-1-carboxylate synthase (ACS), aminocyclopropanecarboxylate oxidase (ACO), and ethylene-responsive transcription factor (ERF), were identified by comparing the DEGs of flower buds at stage III (S4 Table). The expression levels of ACS (CU_locus32671) and ACO (CU_locus22526) were significantly different between long branches and spur twigs (Fig 7). Additionally, there was one gene, cytochrome P450 CYP71A1 (CU_locus30850), whose expression levels were also significantly high in the three stages of long branches. CYP71A1 encodes abscisic acid (ABA) hydroxylase, which is essential for regulating embryo germination [25].

Most of these DEGs exhibited highly significant upregulation in the late flower development of long branches. In addition to ERF transcription factors, some DEGs encoding basic helix-loop-helix (bHLH), WRKY, and NAC transcription factors, as well as calcium channel proteins (CML), were also identified in the flower buds of long branches (Fig 7 and S4 Table). For example, the WRKY family was represented by 10 DEGs that were upregulated in long branches.

### DEGs involved in cellulose biodegradation

**Fig 8:**
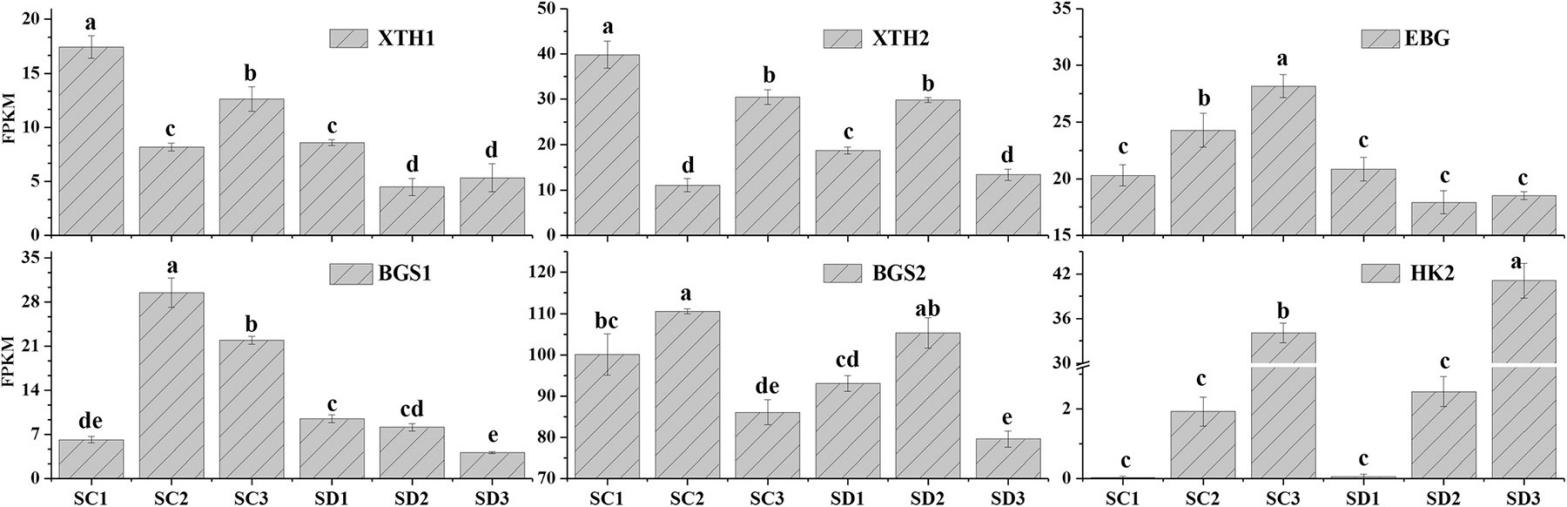
Expression analyses of genes related to cellulose degradation. The x-axis indicates the different stages. The y-axis shows the expression values of the FPKMs. All values were repeated three times and the data are presented as the mean±SD. Different letters indicate significant differences at P<0.01. XTH1: CU_locus1414; XTH2: CU_locus1411; EBG: CU_locus7296; BGS1: CU_locus37299; BGS2: CU_locus24729; HK2: CU_locus39380.

The abortive pistil disappears because of autolysis or atrophy in PCD. Cellulose is a major component of the cell wall. Endoglucanase (EBG), also called cellulase, is responsible for the glycosylation of cellulose. β-Glucosidase (BGS) is responsible for the further hydrolysis of cellulose disaccharides and cellulose oligosaccharides to produce glucose. Xyloglucan endotransglucosylase/hydrolase (XTH) is believed to play an important role in the relaxation and reconstruction of cell walls [26]. In our data set, a total of five genes related to cell wall breakdown were identified (S4 Table), and among these genes were one EBG enzyme gene (CU_locus7296), two BGS enzyme genes (CU_locus37299 and CU_locus24729), and two XTH enzyme genes (CU_locus1411 and CU_locus1414). The expression levels of these genes in flower buds were significantly upregulated in long branches (Fig 8).

However, there were the hexokinase (HK) and phosphoglucomutase (PGM) genes catalyzing D-glucose production of sucrose, which showed no significant difference in the expression level of flower buds at stage III between long branches and spur twigs (S2 Fig). This may be related to the fact that they are also involved in the starch breakdown pathway.

### Changes of genes related to glucose metabolism during the late flower bud development

**Fig 9:**
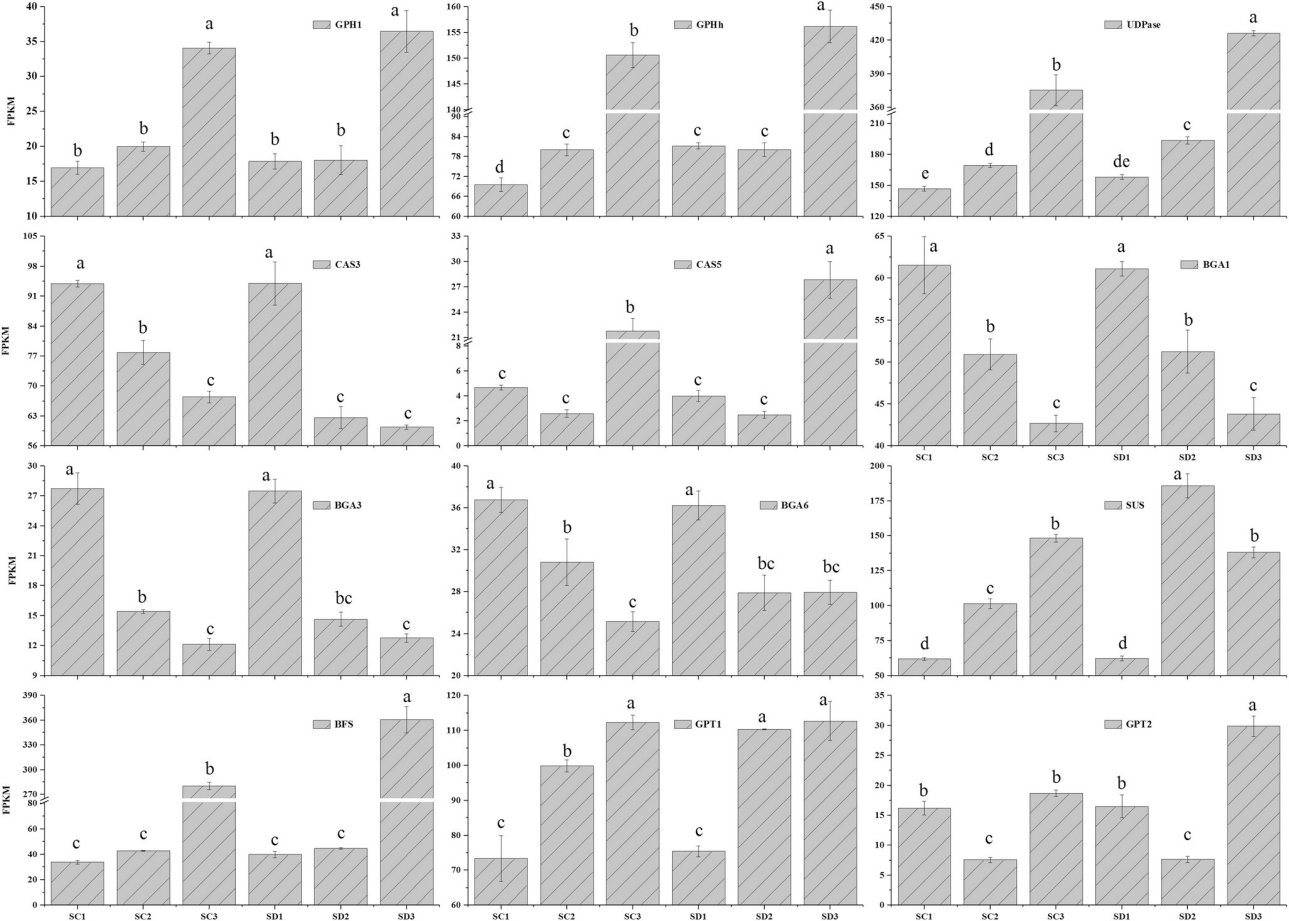
Expression analyses of genes related to starch breakdown, sucrose synthesis and transportation. The x-axis indicates the different stages. The y-axis shows the expression values of the FPKMs. All values were repeated three times and the data are presented as the mean±SD. Different letters indicate significant differences at P<0.01. GPHl: CU_locus25159; GPHh: CU_locus35718; UDPase: CU_locus18418; CAS3: CU_locus34862; CAS5: CU_locus29673; BGA1: CU_locus24690; BGA3: CU_locus7299; BGA6: CU_locus43; SUS: CU_locus46184; BFS: Glucosucrase, CU_locus32011; GPT1: CU_locus27685; GPT2: CU_locus24728.

Storage starch covers long-term carbon needs, fueling germination, or regrowth after certain dormancy periods [27]. Glycogen phosphorylase (GPH), also called starch phosphorylase, catalyzes the first step in the degradation of starch branches, and glucose-1-phosphate (D-gluc-1P) is then converted to UDP-glucose by UDP-glucose pyro-phosphorylase (UDPase), which is then converted to D-glucose, which is finally synthesized into sucrose under the action of two enzymes: callose synthase (CAS) and 1,3-β-D-glucan glucanohydrolase (BGA). The expression of genes related to starch breakdown showed a gradually increasing trend in the development of flower buds of both long branches and spur twigs (Fig 9), indicating that the growth of flower buds requires a large amount of carbohydrates to provide energy.

The flower bud is a heterotrophic tissue that requires the turnover and transport of starch from other tissues to the growing zone. We also identified a large number of highly expressed sugar transport genes (Fig 9) and transitory starch synthase genes (S2 Fig) during late flower bud development. The expression of these genes in flower buds increases as flower buds develop after awakening. HK plays an important role in the sugar pathway producing Glucose-6-phosphate, which is translocated to plastids by the Glucose-6-phosphate transporter (GPT). PGM converts Glucose-6-phosphate to Glucose-1-phosphate. Then, Glucose-1-phosphate is converted to UDP-glucose by UDPase. UDP-glucose is then metabolized to sucrose by sucrose synthase (SUS).

### Validation of genes related to pistil abortion by qRT-PCR

**Fig 10:**
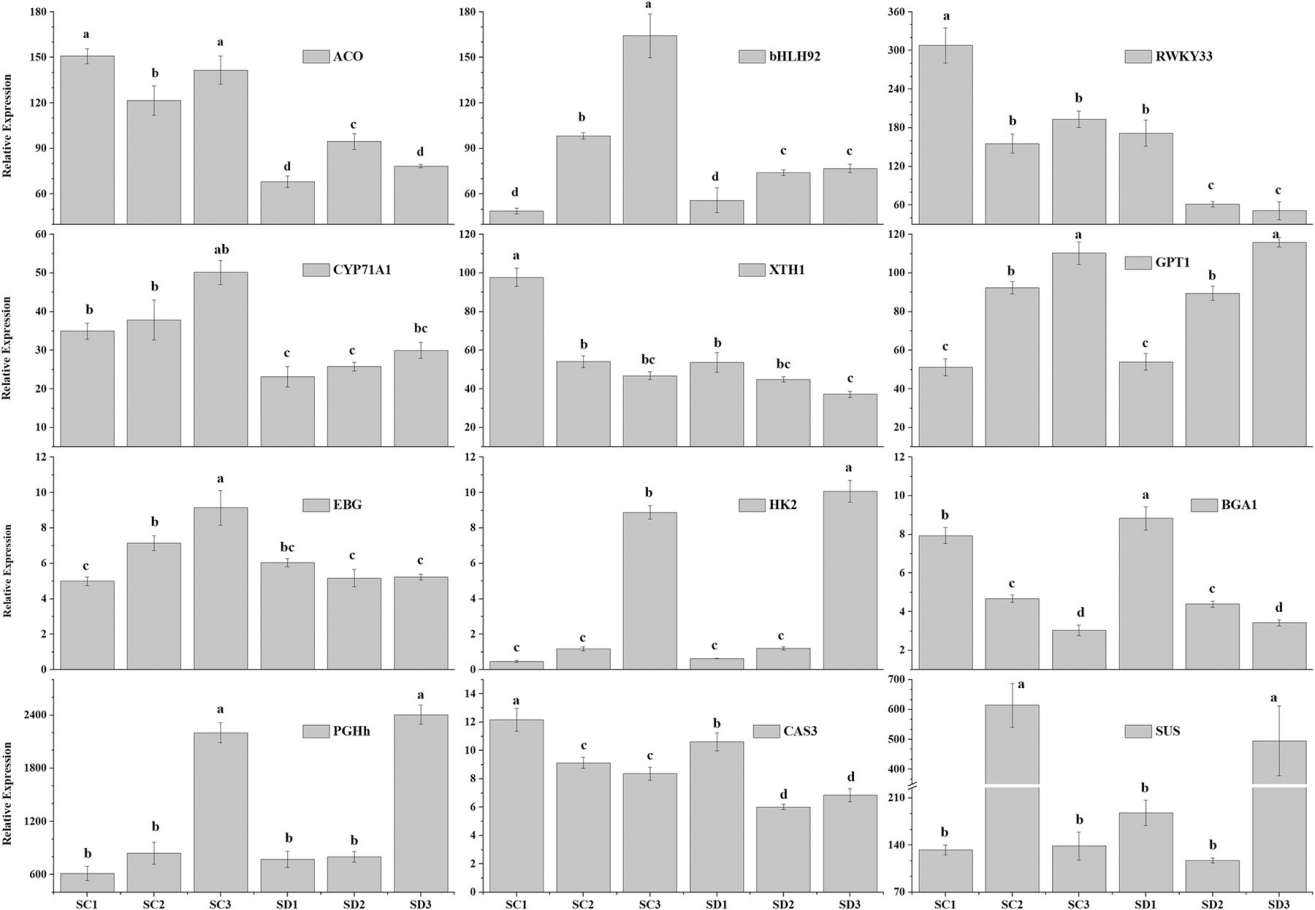
Verification of the selected genes related to the pistil abortion by qRT-PCR. The x-axis indicates the different stages. The y-axis shows the qRT-PCR expression values. The qRT-PCR values were determined by the actin (ACT) gene as an internal reference using the 2-ΔΔCT values. All values were repeated three times and the data are presented as the mean±SD. Different letters indicate significant differences at P<0.01. ACO: CU_locus22526; CYP71A1: CU_locus30850; WRKY33: CU_locus40517; bHLH92: CU_locus476; XTH1: CU_locus1414; EBG: CU_locus7296; HK2: CU_locus39380; GPHh: CU_locus35718; CAS3: CU_locus34862; BGA1: CU_locus24690; SUS: CU_locus46184; GPT1: CU_locus27685.

To evaluate the validity of the RNA-seq data and further confirm the identified differential gene expression patterns, Real-time qRT-PCR was used to estimate the gene expression of 12 randomly selected from RNA-seq data. As shown in Fig 10, these genes included hormones, transcription factors, cell wall degradation, sugar metabolism, and transport-associated genes. The expression level of the ACS gene in long branches was higher than that in spur swig. The expression of CYP71A1, bHLH29, and EBG genes were mainly upregulated during later flower bud development, and the expression level of these genes in long branches was higher than that in spur swig. The expression of WRKY33, XTE, and CAS3 genes were downregulated during the later flower bud development, and the genes were significantly higher expressed in long branches compared with that in spur swig. The expression levels of GPHh, GPT1, and HK1 genes were significantly increasing during the later flower bud development, and the genes were not significant between long branches and spur swig. The expression levels of the BGA1 gene were significantly downregulated during the later flower bud development. The relative expression levels of these selected genes were consistent with the trends in the RNA-seq data (Fig 10).

## Discussion

It is well known that fruit trees entering adulthood are always in a balance between vegetative and reproductive growth. In apricots, spur twigs or long branches can easily obtain flower buds; however, spur twigs have high reproductive capacity, easily bear fruit, and while long branches are of poor quality and low fruit setting. Carbohydrate and other nutrient storage are the most well-known factors involved in flower quality. The low quality of flowers is characterized by either pistils below the stames, withered pistils, pistil hypotrophy, or necrosis. Pistil abortion frequently occurs in apricots and has seriously affected fruit production in many apricot cultivars. Many studies have shown that besides temperature [11, 28] and temperature-induced physiological dormancy [6], nutrient storage [29] was also an essential internal factor in pistil abortion. However, the molecular mechanisms of nutrient storage and flower abortion are still not fully understood in apricots. Here, based on cytological and transcriptome sequencing results, we preliminarily discussed the molecular mechanism of pistil abortion and carbohydrate supply during late flower bud development in apricots.

### A critical stage of pistil abortion in apricots

Flower buds in apricots grew and developed from early differentiation up to dormancy, and resumed growth after dormancy following a continuous development pattern; however, the majority of bud development in long branches did not develop pistil necrosis [30]. The imperfect flowers are characterized by either pistils below the stamens, withered pistils, or an absence of pistils, and such flowers fail to bear fruit. While most of the flowers in the spurs or short twigs at blooming (57%) had a morphologically well-developed pistil, more than 83% of the flowers at blooming presented pistils dysplasia in the long branches, with an underdeveloped or withered pistil in the ‘Shajinhong’ cultivar [7]. Shi et al. [2] indicated that flower buds did not continue to elongate; instead, pistil differentiation stagnated and gradually disintegrated during mid-December, which was the key stage of pistil abortion in Japanese apricots. Two time waves of the pistil abortion were identified during the stages from mid-November to mid-December and from mid-February to mid-March of the following year in the kernel-using apricot [11]. Pistil abortion of ‘Yubada’ cultivars might have occurred during the initial protrusion of the petals stage [10]. In this study, the pistil abortion rates of long branches and spur twigs were similar in the first two stages (at the I and II stages); however, the pistil abortion rates of long branches increased rapidly at the III stage in ‘Shajinhong’ apricots. Therefore, this result suggests that the critical time of pistil abortion was at the III stage (separation of bud scales).

### PCD in pistil abortion

Abnormally underdeveloped pistils are generally caused by the abortion of flower primordia due to cellular necrosis [31]. Necrosis or senescence of local organs in plants is a typical case of PCD [32]. In this study, 163 DEGs were identified in the transcriptome database during the critical stage of abortive flower development between long branches and spur twigs (S4 Table), which were associated with metabolism, signal transduction, and member transport. These transcripts possibly encode genes that respond to pistil abortion or PCD. Pistil abortion is a process in which organ development is halted and cells aging [2, 24]. Cysteine protein involved in the aging process, while cysteine and aspartic proteinases are essential proteolytic enzymes involved in pistil necrosis [33]. Here, we identified four genes encoding cysteine protease (CU_locus39083, CU_locus39087, CU_locus24846, and CU_locus6071), two genes encoding aspartic proteinase (CU_locus9435 and CU_locus48526) that were uniformly upregulated in stage III in long branches. Similar results have been reported in the transcriptomic analysis of chrysanthemum embryos [34].

After the initiation of PCD, the expression of many genes, including transcription factors (TFs), hydrolases such as ribonuclease and protease, and enzymes for ethylene synthesis. PCD has two critical features, including cytochrome C released from the mitochondria into the cell, and the completed DNA chain decomposes into small fragments under the action of endonuclease. The PCD process of pistil cells also demonstrated the release of cytochrome C [35]. In this study, we found one endonuclease (CU_locus46331) and five encoding cytochrome P450 genes (CU_locus24570, CU_locus17804, CU_locus37437, CU_locus30850, and Pa_newGene_114), which were upregulated in the III stage of long branches. Therefore, we believe that PCD occurred in pistil abortion during late flower development.

### Changes in plant hormones and transcription factor involved in PCD during pistil abortion

The occurrence of pistil abortion is usually accompanied by significant changes in endogenous hormones in reproductive organs [36]. Previous studies have established a connection between plant hormones and pistil abortion at the physiological level, but the underlying mechanism still needs further research. ETH is an important regulatory hormone that initiates PCD. Target cells, which are extremely sensitive to low concentrations of endogenous ETH, produce cellulase and other polysaccharide-degrading enzymes, which are secreted into the cell wall and cause cell wall relaxation [24]. In this study, we detected two key genes involved in ethylene synthesis (ACS and ACO) and ERF genes of the ethylene reaction element binding protein family (CU_locus10116, CU_locus32454, CU_locus17740, CU_locus45365, and CU_locus50531) were significantly highly expressed in the flower buds of long branches. The high expression of those genes might result in the degradation of the cell wall in the pistil. Andreini et al. [6] also found that the necrotic or abscission region in flower buds was characterized by bands of smaller thin-walled cells susceptible to rupture, which led to the formation of extensive intercellular spaces. ABA is an important signal that converts abiotic environmental factors into the regulation of flower development and promotes or represses flowering, independent of the florigen genes [37]. Oliver et al. [38] reported that exogenous ABA treatment at the young microspore stage induced pollen sterility, and excessive ABA production had caused the degeneration and death of pollen mother cells [39]. Our data also showed that the expression levels of the CYP707A1 gene in spur twigs, which was considered to play a predominant role in ABA catabolism [40], were significantly lower than those in long branches. In Arabidopsis, expression analysis also indicated that CYP707A1 was responsible for the rapid decrease in ABA levels during embryo germination [25]. These results demonstrate that ETH and ABA play critical regulatory roles in pistil abortion during the late flower bud development in apricots.

Ca2+ and transcription factors are also involved in the signal transduction pathway of PCD [41]. Our transcriptome database showed that the six genes encoding CML or transporting proteins (CU_locus33375, CU_locus24324, CU_locus22748, CU_locus361, CU_locus363, and CU_locus41204), transcription factors such as bHLH (CU_locus33758 and CU_locus476), and WRKY (CU_locus8051, CU_locus16052, and CU_locus8596) were significantly upregulated in long branches. Moreover, except for the key genes involved in pistil abortion, some genes encoding serine/threonine protein kinase were highly expressed in stage III, such as CU_locus51031, CU_locus51236, and CU_locus10380. Therefore, we speculated that these differential genes might also play an important role in the pistil abortion of long branches in apricots.

### Cellulose degradation in cell wall related to pistil abortion

PCD is the programmed degradation of cellular contents that leads to the re-transport of nutrients to benefit future developmental processes [41]. Ethylene signaling induced the transcriptional expression of cellulase and polysaccharide-degrading enzyme genes, causing cell wall relaxation [42]. XTH catalyzes the cleavage and molecular grafting of xyoglucan chain functions in the loosening of the cell wall and is believed to play an important role in relaxation and reconstruction [26]. The expression of XTH2 might be related to pistil abortion in Japanese apricots [2]. In this study, two XTH genes were significantly higher in stage III of long branches than in stage II or spur twigs.

Cellulose is the main polysaccharide component of cell walls. Under the action of XTH, the cellulose in the loose cell wall is converted by cell-wall degrading enzymes, such as BGS, EBG, and polygalacturonase, eventually to D-glucose. Under the catalysis of HK, PGM, UDPase, and SS, glucose is converted into sucrose, which is transported to the growing point [26]. A total of four difference genes (S4 Table) were identified between SD3vsSC3 and SC2vsSC3 in the cellulose metabolism pathway, including two BGS enzyme genes, one EBG enzyme gene, and one polygalacturonase gene (CU_locus11171). However, the levels of HK, PGM, UDPase, and SS genes were not significantly increased in the flower buds of long shorts. This might be related to the fact that these enzymes are also involved in starch metabolism in the flower buds of spur twigs. During specific phases of development or periods of sugar starvation, some of the polysaccharides in the cell wall may be hydrolyzed into their constituent sugars to be scavenged by the cell and used to meet the cell’s needs [24]. Pistil abortion is a specialized type of PCD, which releases β-1,3-glucanase to hydrolyze cellulose in the loose cell wall [43]. PCD could provide subsequent substances applied to growing zones in the case of carbohydrate deficit. Therefore, we speculated that cell wall degradation plays a key role in ovary visible atrophy due to insufficient carbohydrate supply.

### Differences in carbohydrate transport between long branches and spur twigs after dormancy release

After the dormancy of deciduous fruit trees, the tree body begins a new annual cycle of growth, and the adult tree requires a lot of carbohydrates for reproduction and vegetative growth [4, 6]. The flower buds of spur twigs were significantly heavier than buds on long branches during bud break and the following stages of development before blooming [4], showing that the competition for nutrients between buds and growing branches could be behind these alterations in different types. Starch is the main storage carbohydrate nutrient of plants, and its accumulation in the pistil plays a clear role in the support of pollen tube growth [12] and ovule fate [13]. At this point, it is very active for carbohydrate metabolism in heterotrophic tissue turnover and transport into new points. In this study, we analyzed transcriptome data of different stages of flower buds between long branches and spur twigs, and related the transcriptional dynamics to the observed pistil abortion.

Storage starch covers long-term carbon needs, fueling blooming, or regrowth after certain dormancy periods [27]. However, starch synthesis in heterotrophic tissues, such as flower organs, relies on the translocation of carbon from other tissues, mainly in the form of hexose phosphate [44]. Transitory starch is considered important for flower and early embryo development. GPT acted as the putative translocator of Glc-6-phosphate for starch biosynthesis in reproductive tissues [45]. Clément et al. [46] found that one–two cycles of starch biosynthesis occur either in the sporogenous cells or the anther wall layer, and the third starch wave during the last stages of stamen development was also identified in Arabidopsis thaliana [45]. Before blooming, flower bud development requires a large amount of carbohydrate nutrients. In this study, it is continuously increased that the genes expression of carbohydrate metabolism activity, including starch breakdown and sugar transport and new starch synthesis, among the three stages of flower development. In addition, the expression levels of EBG and BGS genes related to cellulose gradually increased along with ovary atrophy. During the last stages of unwilled flower development, while the progressive depletion of starch was observed in the placenta-septum region, conspicuous starch deposition occurred in both styles [45]. Although starch accumulation does not represent a carbohydrate translocation process, correlations in starch turnover between adjacent tissues or specific patterns of starch accumulation suggest potential starch degradation, sugar translocation, and nutrient transport processes. In apricots, it is frequently observed that the pollen of the flower is normal, while the pistil is underdeveloped or abortive in long branches [4]. Therefore, we hypothesized that the EBG and BGS transform cellulose into soluble sugar could replace starch decomposition to provide nutrients for stamens in the case of carbohydrate deficiency, so as to transmit their genes to future generations.

**Fig 11:**
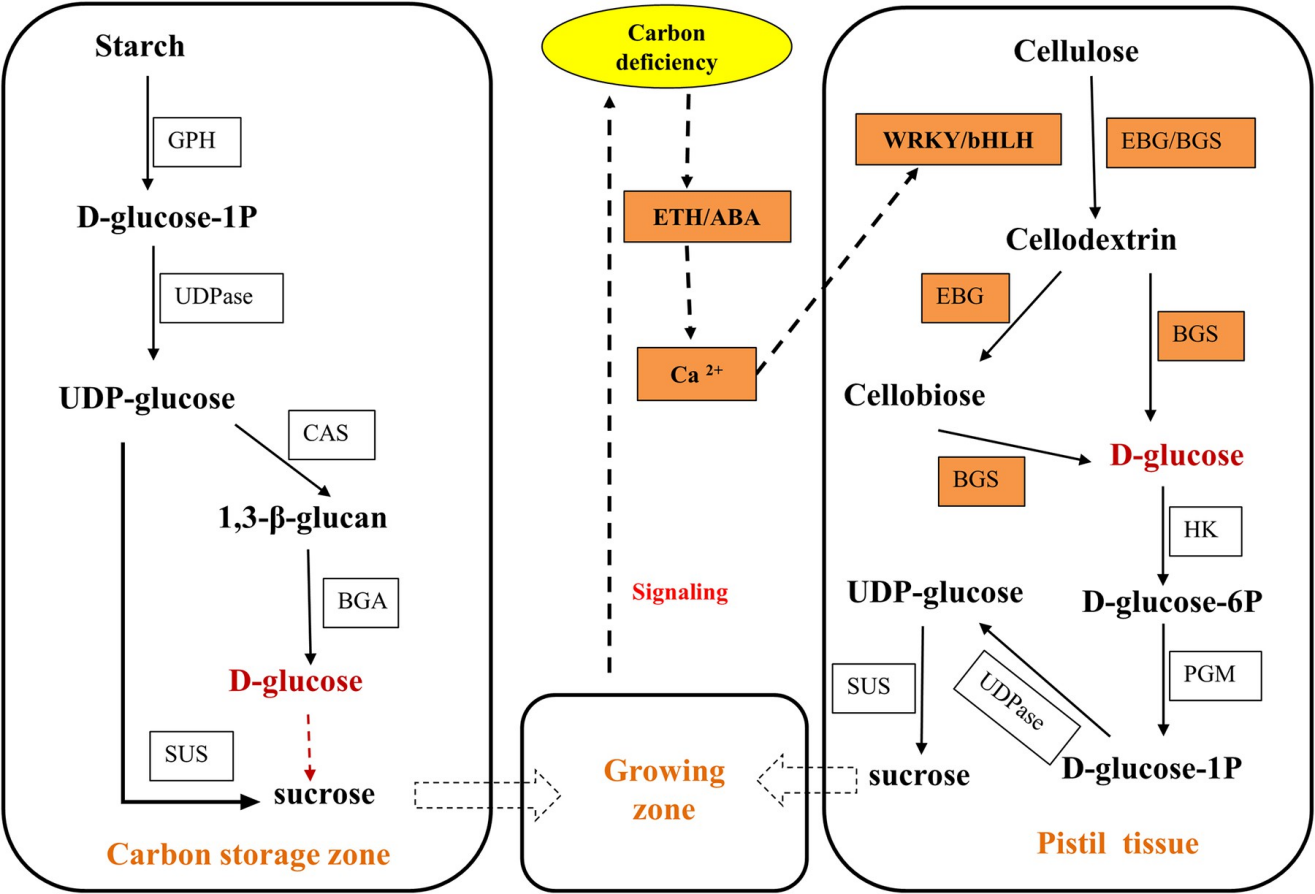
Schematic diagram of the mechanism of pistil abortion in apricots.

In a word, we propose a preliminary hypothesis model that carbon deficiency causes the pistil abortion of flower buds before flowering in long branches. The PCD of the necrotic cell could activate the hormone signal and transcription factors, resulting in cellulose in the cell wall is broken down and converted into sugars, and then transported to other tissues for growth (Fig 11). Although it is unclear the difference in the genes expression and carbohydrate accumulation between pistils and stamens in the same flower, the hypothetical model is presented for the molecular mechanism of pistil abortion in long branches in ‘Shajinhong’ apricot. The mechanisms of pistil abortion in long branches need to be further improved. For example, it is necessary to separate the pistils and stamens, and then test the starch content and quantitative PCR verification; however, as anomaly pistils and well stamens in the same flower bud cannot be separated because of limited experimental techniques.

## Conclusions

In this study, we conducted an experiment on the different cytological and molecular characteristics of flower buds between spur twigs and long branches in apricots. The cell wall degradation and the PCD of the necrotic cell caused by carbon deficiency at stage III (separation of bud scales) result in the pistil abortion of flower buds in ‘Shajinhong’ apricots. This result provides a theoretical basis for fertilization that the rate of flower bud abortion can be reduced by increasing carbohydrate accumulation of branches in the last year. Here, a rudimentary molecular mechanism model was proposed to the pistil abortion of long branches of apricot. In this model, storage nutrient deficiency causes in cell wall decomposition through endogenous hormones and transcription factors, however, the regulation of key genes needs to be further studied. The present studies may provide insights into understanding the pistil abortion during the later flower bud development stage and help us to develop an effective select strategy for high-yield breeding in apricot.

## Supporting information

S1 Fig Weighted gene co-expression network analysis. (TIF)

S2 Fig Expression analyses of genes related to the starch synthesis. (TIF)

S1 Table Primers used to perform qRT-PCR of genes. (XLSX)

S2 Table A total of 478 genes optimized the gene structure. (XLSX)

S3 Table The 1516 novel genes function annotation. (XLSX)

S4 Table A total of 163 differentially expressed genes between long branches and spur twigs. (XLSX)
