# Supplementary figures and images for: Transcriptome analysis of flower bud identified genes associated with pistil abortions between long branches and spur twigs in apricots (Prunus armeniaca L.)
Source: PLoS One. 2022 Aug 26;17(8):e0273109. doi: 10.1371/journal.pone.0273109 (PMC9417009; doi:10.1371/journal.pone.0273109)

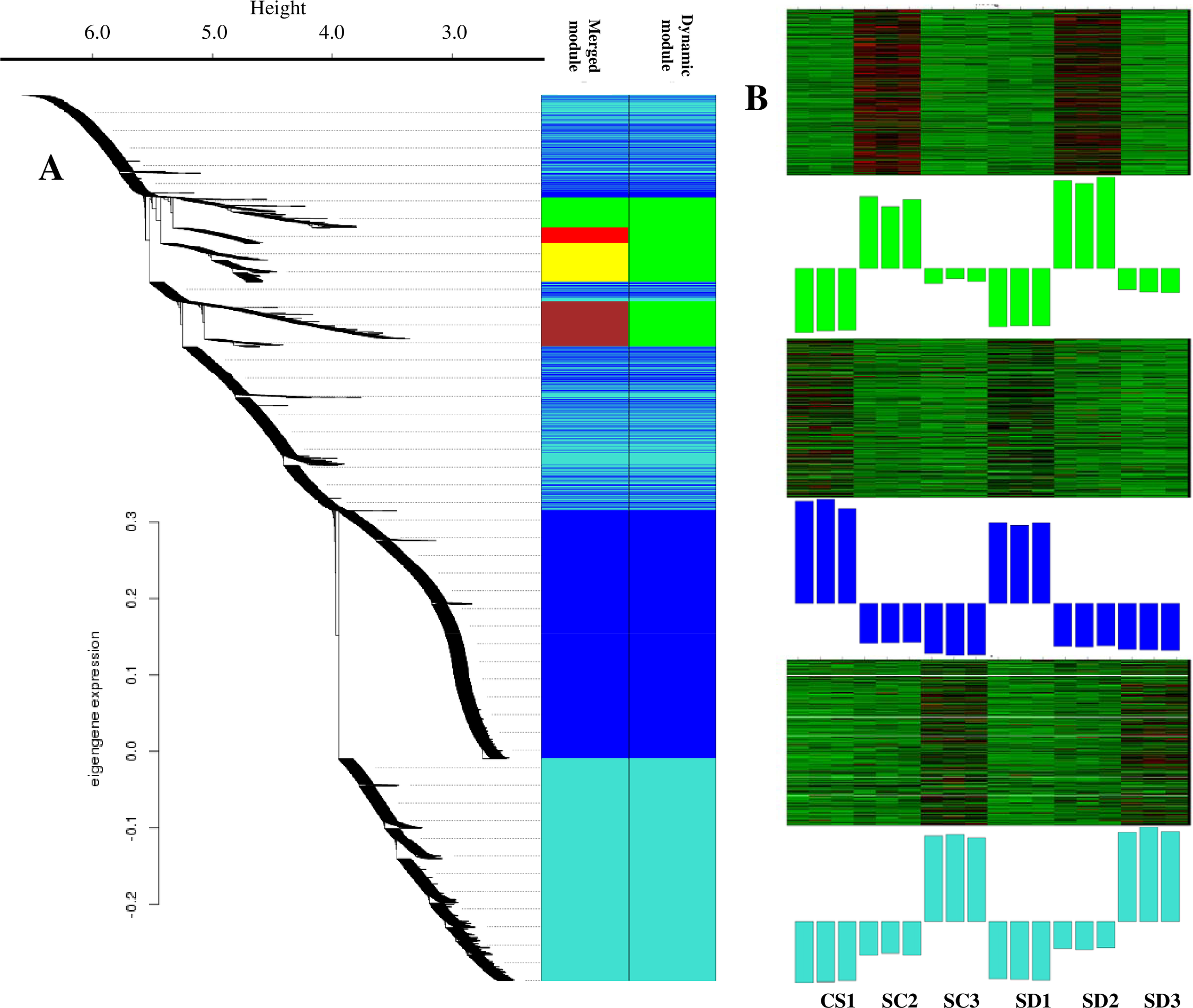

Supplement: S1 Fig — (TIF) [file pone.0273109.s001.tif]

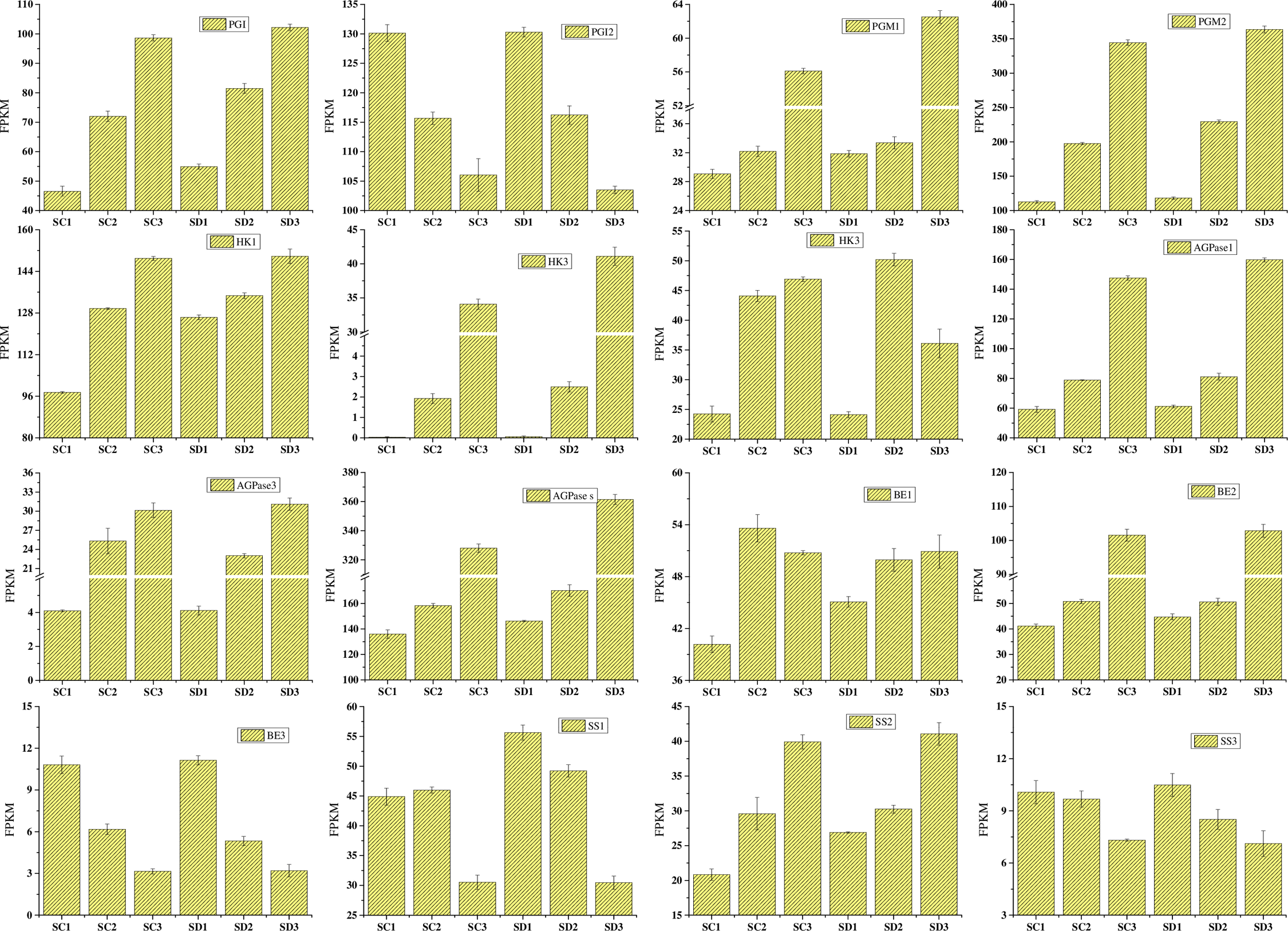

Supplement: S2 Fig — (TIF) [file pone.0273109.s002.tif]
